# Time between Symptom Onset, Hospitalisation and Recovery or Death: Statistical Analysis of Belgian COVID-19 Patients

**DOI:** 10.3390/ijerph17207560

**Published:** 2020-10-17

**Authors:** Christel Faes, Steven Abrams, Dominique Van Beckhoven, Geert Meyfroidt, Erika Vlieghe, Niel Hens, Belgian Collaborative Group on COVID-19 Hospital Surveillance

**Affiliations:** 1 Data Science Institute (DSI), I-BioStat, Universiteit Hasselt, BE-3500 Hasselt, Belgium; steven.abrams@uhasselt.be (S.A.); niel.hens@uhasselt.be (N.H.); 2 Global Health Institute (GHI), University of Antwerp, BE-2000 Antwerp, Belgium; 3 Department of Epidemiology and Public Health, Sciensano, BE-1050 Brussels, Belgium; dominique.vanbeckhoven@sciensano.be (D.V.B.); hospital_datacollection@sciensano.be (B.C.G.o.C.H.S.); 4 Department and Laboratory of Intensive Care Medicine, University Hospitals Leuven and KU Leuven, Herestraat 49, Box 7003 63, 3000 Leuven, Belgium; geert.meyfroidt@kuleuven.be; 5 Department of General Internal Medicine, Infectious and Tropical Diseases, University Hospital Antwerp, BE-2000 Antwerp, Belgium; erika.vlieghe@uantwerpen.be; 6 Centre for Health Economics Research and Modelling of Infectious Diseases (CHERMID), Vaccine & Infectious Disease Institute (VAXINFECTIO), University of Antwerp, BE-2000 Antwerp, Belgium

**Keywords:** COVID-19, length of stay in hospital, symptom onset to hospitalization, truncation and interval-censoring

## Abstract

There are different patterns in the COVID-19 outbreak in the general population and amongst nursing home patients. We investigate the time from symptom onset to diagnosis and hospitalization or the length of stay (LoS) in the hospital, and whether there are differences in the population. Sciensano collected information on 14,618 hospitalized patients with COVID-19 admissions from 114 Belgian hospitals between 14 March and 12 June 2020. The distributions of different event times for different patient groups are estimated accounting for interval censoring and right truncation of the time intervals. The time between symptom onset and hospitalization or diagnosis are similar, with median length between symptom onset and hospitalization ranging between 3 and 10.4 days, depending on the age of the patient (longest delay in age group 20–60 years) and whether or not the patient lives in a nursing home (additional 2 days for patients from nursing home). The median LoS in hospital varies between 3 and 10.4 days, with the LoS increasing with age. The hospital LoS for patients that recover is shorter for patients living in a nursing home, but the time to death is longer for these patients. Over the course of the first wave, the LoS has decreased.

## 1. Introduction

The world is currently faced with an ongoing coronavirus disease 2019 (COVID-19) pandemic. The disease is caused by the severe acute respiratory syndrome coronavirus 2, a new strain of the coronavirus, which was never detected before in humans, and is a highly contagious infectious disease. The first outbreak of COVID-19 occurred in Wuhan, province Hubei, China in December 2019. Since then, several outbreaks have been observed throughout the world. As from 7 March, the first generation of infected individuals as a result of local transmission was confirmed in Belgium.

There is currently little detailed knowledge on the time interval between symptom onset and hospital admission, nor on the length of stay (LoS) in hospital in Belgium. However, information about the LoS in hospital is important to predict the number of required hospital beds, both for beds in general hospital and beds in the intensive care unit (ICU), and to track the burden on hospitals [1]. The time delay from illness onset to death is important for the estimation of the case fatality ratio [2]. Individual-specific characteristics, such as the gender, age and co-morbidity of the individual, could potentially explain differences in LoS in the hospital.

Therefore, we investigate the time of symptom onset to hospitalization and the time of symptom onset to diagnosis, as well as the LoS in hospital. We consider and compare parametric distributions for these event times enabling to appropriately take care of truncation and interval censoring. In Section 2, we introduce the epidemiological data and the statistical methodology used for the estimation of the parameters associated with the aforementioned delay distributions. The results are presented in Section 3 and avenues of further research are discussed in Section 4.

## 2. Methods

### 2.1. Clinical Surveillance of COVID-19 Hospitalized Patients

The hospitalized patients clinical database is an ongoing multicenter registry in Belgium that collects information on hospital admission related to COVID-19 infection. The data are regularly updated as more information from the hospitals are sent in. The individual patients’ data are collected through 2 online questionnaires: one with data on admission and one with data on discharge. Data are reported for all hospitalized patients with a confirmed COVID-19 infection. The reporting is strongly recommended by the Belgian Risk Management Group, therefore the reporting coverage is high (>70% of all hospitalized COVID-19 cases) [3].

**Figure A2:**
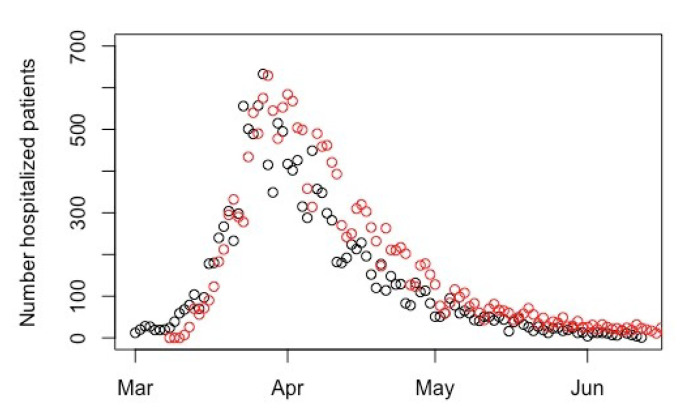
Observed number of new hospitalization in the national surveillance survey (black) and reported number of confirmed hospitalizations in the population (red).

At the time of writing this manuscript, there is information about 14,618 patients, hospitalized between 1 March 2020 and 12 June 2020, including age and gender. Table A1 (Appendix B) summarizes the age and living status (living in nursing home or not) of the patients. Age is categorized into 4 age groups: the young population (0–20 years), the working age population (20–60 years), the senior population (60–80 years) and the elderly (80+ years). It shows that a large proportion of the hospitalized 60+ patients live in a nursing home facility (about 12% for patients aged 60–79 and 35% for patients aged 80+). The survey contains information on 1831 patients hospitalized during the initial phase of the outbreak (between 1 March and 20 March); 4998 patients in the increasing phase of the outbreak (between 21 March and 31 March); 5094 in the descending phase (between 1 April and 18 April); and 2695 individuals at the end of the first wave of the COVID-19 epidemic (between 19 April and 12 June). The time trend in the number of hospitalizations is presented in Figure A2 (Appendix B). The time trend in the survey matches well with the time trend of the outbreak in the whole population, though with some under-reporting in April and May.

The time variables (time of symptom onset, hospitalisation, diagnosis, and recovery or death) were checked for consistency. Observations identified as inconsistent were excluded for analyses. Details of the inclusion and exclusion criteria are provided in Appendix A. Some descriptive analyses of the event times are provided in Appendix C.

### 2.2. Statistical Model

Different flexible parametric non-negative distributions can be used to describe the delay distributions, such as the exponential, Weibull, lognormal and gamma distributions [4]. However, as the reported event times are expressed in days, the discrete nature of the data should be accounted for. Reference [2,5] assume a discrete probability distribution parameterized by a continuous distribution. Alternatively, Reference [6] estimate the serial interval using interval censoring techniques from survival analysis. Reference [7,8] use doubly interval-censoring methods for estimation of the incubation distribution. We use interval-censoring methods originating from survival analysis to deal with the discrete nature of the data, to acknowledge that the observed time is not the exact event time [9]. Let $x_{i}$ be the recorded event time (e.g., LoS in hospital). Instead of assuming that $x_{i}$ is observed exactly, it is assumed that the event time is in the interval $\left(L_{i},R_{i}\right)$, with $L_{i}=x_{i}-0.5$ and $R_{i}=x_{i}+0.5$ for $x_{i}\geq 1$ and $L_{i}=\epsilon =10^{-3}$ and $R_{i}=0.5$ for $x_{i}=0$. As a sensitivity analysis, we compare this assumption with the wider interval $\left(L_{i}=x_{i}-1,R_{i}=x_{i}+1\right)$.

An additional complexity is that the delay distributions are truncated, either because there is a maximal clinical delay period or because the hospitalization is close to the end of the study. First, only patients reporting a delay between symptoms and hospitalization (or diagnosis) of at most 31 days were included in the study, because it is unclear for the other patients whether the reason for hospital admission was COVID-19 infection. In literature, times from onset of symptoms to hospital admission have been reported between 4 and 15 days (e.g., Reference [10,11,12,13]), with no mention of observed delay times above 31 days. Second, if hospitalization is e.g., 14 days before the end of the study, the observed LoS cannot exceed 14 days. However, it has to be noted that only patients that have left the hospital are included in the survey, and as a result it will not include patients that are hospitalized near the end of the survey and have a long length of stay. This is a clear example of right-truncation (as opposed to right-censoring under which patients are still part of the study/data and only partial information is available on their length of stay). We therefore use a likelihood function accommodating the right-truncated and interval-censored nature of the observed data to estimate the parameters of the distributions [6]. The likelihood function is given by

$$L\left(\Theta ;x_{1},\ldots ,x_{N}\right)=\prod_{i}{\left(\frac{F\left(R_{i}|\Theta \right)-F\left(L_{i}|\Theta \right)}{F(T_{i}|\Theta )}\right)}^{w_{i}}$$

in which $T_{i}$ is the (individual-specific) truncation time and F(·) is the cumulative distribution function corresponding to the density function f(·). We truncate the time from symptom onset to diagnosis and the time from symptom onset to hospitalisation to 31 days ($T_{i}\equiv 31$). The LoS in hospital is truncated at $T_{i}=E-t_{i}$, in which $t_{i}$ is the time of hospitalization and *E* denoted the end of the study period (6 June 2020). In addition, to account for possible under-reporting in the survey, each likelihood contribution is weighted by the post-stratification weight $w_{i}\equiv w_{t}$ defined as $w_{t}=\frac{N_{t}}{n_{t}}\sum_{t}n_{t}$, where *t* is the day of hospitalization for patient *i*, $N_{t}$ the number of hospitalizations in the population on day *t* and $n_{t}$ is the number of reported hospitalizations in the survey on day *t*. This weighted likelihood is also called pseudo-likelihood in the context of complex survey data, for which consistency and asymptotic normality has been shown [14].

We assume Weibull and lognormal distributions for the delay distributions. The two parameters of each distribution are regressed on age, gender, nursing home and time period (as well as interactions of these). By assuming both parameters to be covariate-dependent, we allow that both the mean and the range of the time to event variable varies in different population groups. The BFGS optimization algorithm is used to maximize the likelihood. Convergence is reached for all considered models. The Bayesian Information Criterion (BIC) is used to select the best fitting parametric distribution and the best regression model among the candidate distributions/models. Only significant covariates are included in the final model.

## 3. Results

### 3.1. Symptom Onset to Hospitalization and to Diagnosis

**Figure 1:**
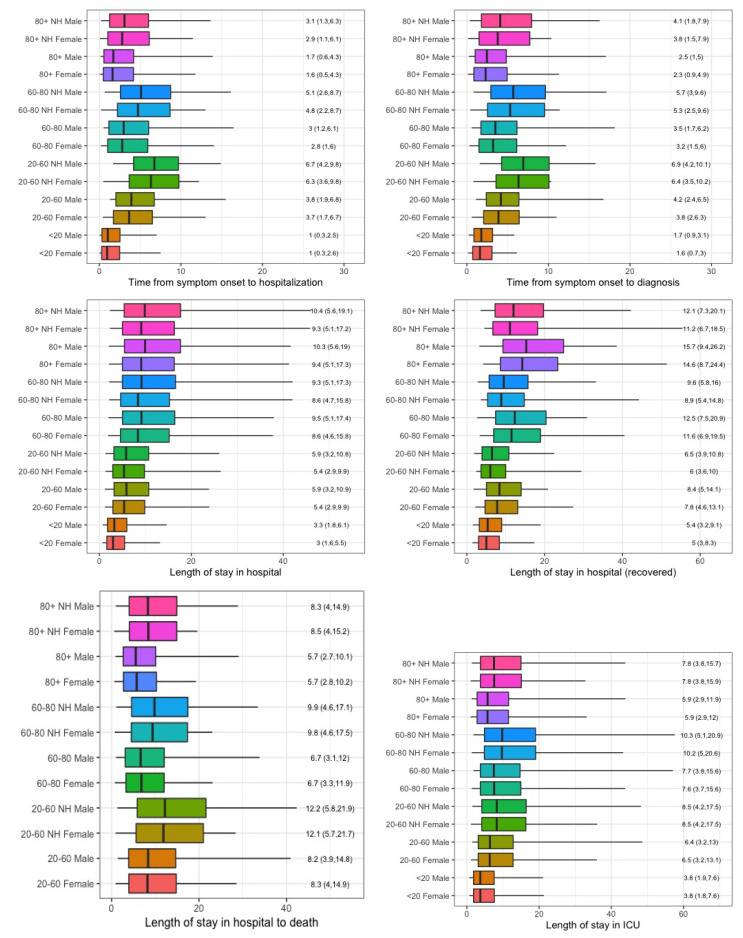
Comparison of delay distribution for different population groups. Top: delay from symptom onset to hospitalization and diagnosis; Middle: LoS for all patients and for recovered patients; Bottom: LoS for patients that died and LoS in ICU. The boxplots show the estimated 5%,25%,50%,75% and 95% quantiles. Results are based on period 21 March to 30 March. The reported values correspond to the 50%(25%,75%) quantiles.

Overall, the delay between symptom onset and hospitalization can be described by a truncated Weibull distribution with shape parameter 0.845 and scale parameter 5.506. The overall average delay is very similar to the one obtained by [15], based on a stochastic discrete time model relying on an Erlang delay distribution. However, there are significant differences in the time between symptom onset and hospitalization amongst different gender groups, age groups, living status and time period of hospitalization. As the truncated Weibull distribution has a lower BIC as compared to the lognormal distribution (66,923 and 68,657 for Weibull and lognormal distributions, respectively), results for the Weibull distribution are presented. In Table 1, the regression coefficients of the scale (λ) and shape parameters (γ) of the Weibull distribution are presented. The impact on the time between symptom onset and hospitalization is visualized in Figure 1, showing the model-based 5%,25%,50%,75% and 95% quantiles of the delay times.

Age has a major impact on the delay between symptom onset and hospitalization, with the youngest age group having the shortest delay (median of 1 day, but with a quarter of the patients having a delay longer than 2.6 days). The time from symptom onset to hospitalization is more than doubled in the working age (20–60 years) and ageing (60–80 years) population as compared to this young population (median close to 4 days and a delay of more than 6.7 days for a quarter of the patients). In contrast the increase is 50% in the elderly (80+ years) as compared to the youngest age group (median delay of 1.6 days, with a quarter of the patients having a delay longer than 4.3 days). After correcting for age, it is observed that the time delay is somewhat higher when patients come from a nursing home facility, with an increase of approximately 2 days. Note that in the descriptive statistics, we observed shorter delay times for patients coming from nursing homes. This stems from the fact that 80+ year old’s have shorter delay times as compared to patients of age 20–79, but the population size in the 80+ group is much larger as compared to the 20–79 group in nursing homes. And although statistical significant differences were found for gender and period, we observe very similar time delays between males and females and in the different time periods (see Figure A7). The differences occur in the tails of the distribution; with, e.g., the 5% longest delay times between symptoms and hospitalizations observed for males.

The time between symptom onset and diagnosis is also best described by a truncated Weibull distribution (shape parameter 0.900, scale parameter 5.657). As again the truncated Weibull distribution has a lower BIC value as compared to the lognormal distribution (68,106 and 69,652 for Weibull and lognormal, respectively), results for the Weibull distribution are presented. Parameter estimates are very similar to the distribution for symptom onset and hospitalization (Table 1). The median delay between symptom onset and diagnosis is approximately one day longer as compared to the median delay between symptom onset and hospitalization. The time from symptom onset to diagnosis in males had a much wider range as compared to females. This is observed in the tails of the distribution, with the 5% longest delay times being 5 days longer for males as compared to females. Especially at the increasing phase of the epidemic, the time between symptom onset and diagnosis was longer as compared to the time between symptom onset and hospitalization (see Figure A7), but this delay has shortened over time.

**Figure A6:**
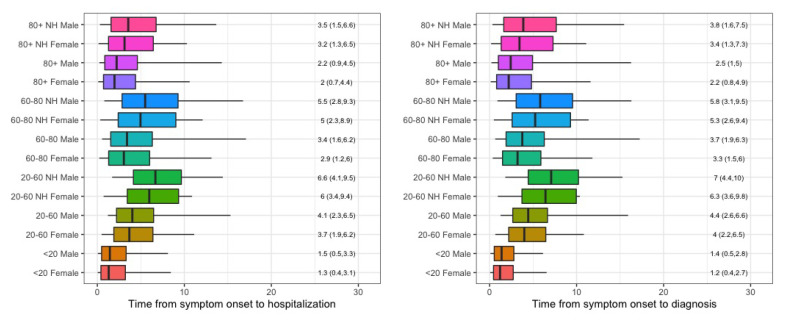
Comparison of delay distribution for different population groups. The boxplots show the estimated 5%,25%,50%,75% and 95% quantiles, based on a truncated Weibull regression. The time intervals are assumed interval-censored in intervals $\left(x_{i}-1,x_{i}+1\right)$. Results are based on period 21 March to 30 March.

**Figure A7:**
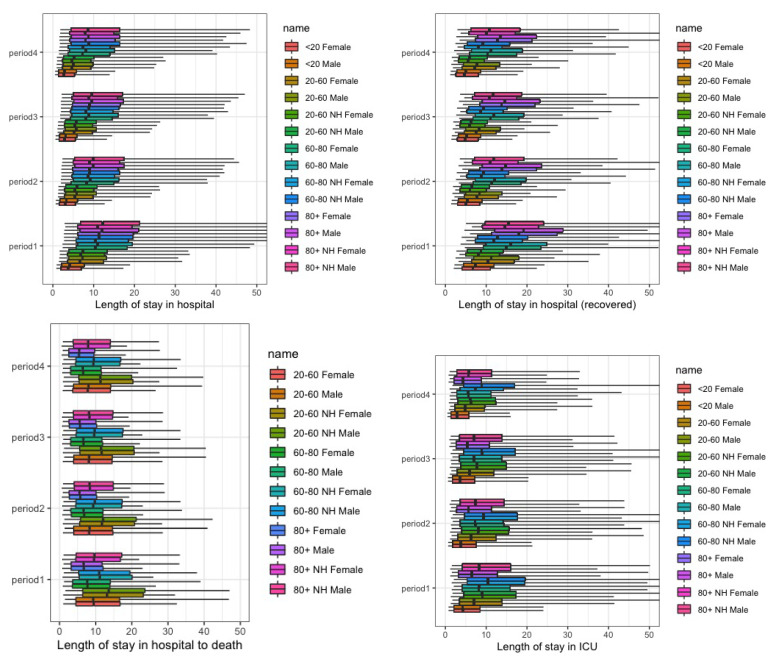
Comparison of delay distribution for different population groups. The boxplots show the estimated 5%,25%,50%,75% and 95% quantiles, based on Weibull regression. Results are based on the time period 01 March to 20 March (period 1), 21 March to 30 March (period 2), 31 March to 18 April (period 3) and 19 April to 12 June (period 4).

**Figure A8:**
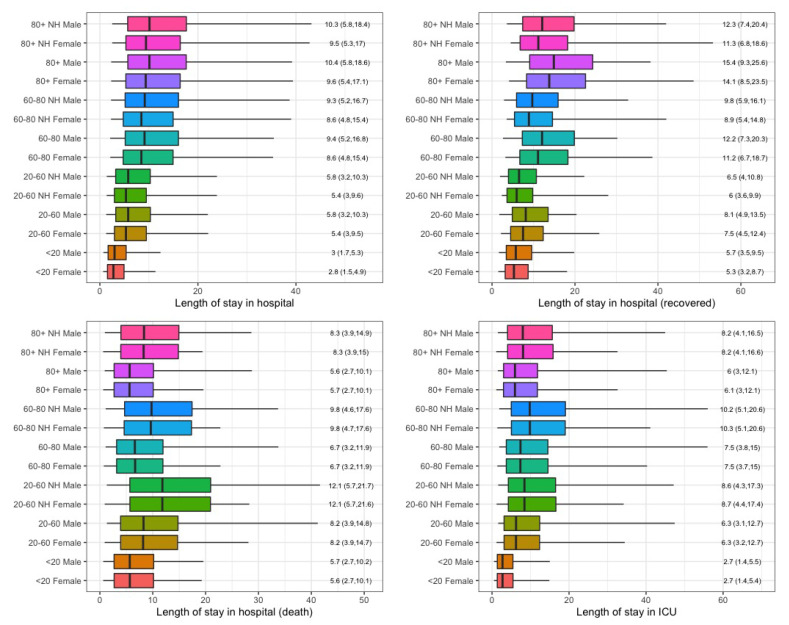
Comparison of delay distribution for different population groups. The boxplots show the estimated 5%,25%,50%,75% and 95% quantiles, based on lognormal regression. The time intervals are assumed interval-censored in intervals $\left(x_{i}-1,x_{i}+1\right)$. Results are based on period 21 March to 30 March.

To test the impact of some of the model assumptions, a comparison is made with an analysis without truncating the time between symptom onset and hospitalisation or diagnosis and wider time intervals $\left(x_{i}-1,x_{i}+1\right)$. Results are presented in Figure A6 and Figure A8, and are very similar to the once presented here. It was also investigated whether or not there a difference between neonati (with virtually no symptoms, but diagnosed at the time of birth or at the time of the mothers testing prior to labour) and other children. For all children <20 years of age, we found a median time from symptom onset to hospitalization and diagnosis to be 1 and 1.6 days, respectively. If we only consider children >0 years of age, a small increase is found (1.5 (0.5–3.4) days for time to hospitalization and 1.8 (0.7–3.7) for time to diagnosis).

### 3.2. Length of Stay in Hospital

A summary of the estimated LoS in hospital and ICU is presented in Table 2 and Figure 1 based on the lognormal distribution. The lognormal distribution has a slightly smaller BIC value as compared to the Weibull distribution for the LoS in hospital (76,928 for Weibull and 76,865 for lognormal) and for the LoS in ICU (7341 for Weibull and 7312 for lognormal).

The median LoS in hospital is close to 3 days in the youngest age group, but 25% of these patients stay longer than 5.5 (8.6) days in hospital for females (males), and 5% stay longer than 13 (14) days for females (males). The LoS increases with age, with a median LoS of around 5.4 (5.9) days for females (males) in the working age group. A quarter of the patients in age group 20–60 stay longer than 10 days and 5% stays longer than 24 days. This increases for patients above 60 years of age, with a median LoS of around 8.6 (9.4) days for female (male) patients in the senior population group and 9.4 (10.3) days for female (male) patients in the elderly group. A large proportion of the elderly patients stay much longer in hospital. A quarter of these patients stay longer than 15.7–17.4 days for patients in the ageing group and longer than 17.3–19 days for the elderly. Some very long hospital stays are observed in these age groups, with 5% of the LoS being longer than 38 (41) days for females (males) in the ageing group, and 42 (46) days in the elderly. No significant difference is found for patients coming from nursing homes. Over the course of the first wave, the LoS has slightly decreased, with a decrease in median LoS of around 2 days from the first period to later periods. Note that this result is corrected for possible bias of prolonged lengths of stay being less probable for more recently admitted patients.

The LoS in ICU (based on the lognormal distribution) is on average 3.8 days for the young patients, with a quarter of the patients staying longer than 7.6 days in ICU. Similar to LoS in hospital, also the LoS in ICU increases with age. The median LoS in the working age population is 6.4, in the senior population 7.6, while in elderly it is slightly shorter (5.9 days). Again, it is observed that a quarter of the patients in age group 20–60 stay longer than 13 days in ICU, in age group 60–80 15.6 days and in 80+ 12 days. Patients living in nursing homes stay approximately 2 days longer in ICU. No major difference is observed in the LoS in ICU between males and females, though some prolonged stays are observed in males as compared to females. Similar as the overall LoS in hospital, the LoS in ICU has decreased over time (with a decrease of 1 day from the first period to the later periods, and an additional 2 days in the last period).

Table 3 summarizes the LoS in hospital for patients that recovered or passed away. The lognormal distribution has the smallest BIC value for time from hospitalization to recovery and the Weibull distribution for time from hospitalization to death. For patients that recovered, the LoS in hospital increased with age (the median LoS is 5 days for the young population, which increases to 8 days in working age population, 12 days in the senior population and 15 days in the elderly). In contrast to previous results, we observe that patients living in nursing homes leave hospital approximately 1 day faster as compared to the general population. However, in contrast, the 5% longest stays in hospital before recovery are longer for patients living in nursing homes.

But, while the LoS in hospital for patients that recover increases with age for all age groups, the survival time of hospitalized patients that died is lower for the age groups seniors (median time of 6.7 days) and elderly (median time of 5.7 days) as compared to the working age group (median time of 12.1 days). Also large differences are observed amongst patients coming from nursing homes or not, with the time between hospitalization and death being approximately 3 days longer for patients living in a nursing home. No significant differences are found between males and females.

A sensitivity analysis assuming that the time delay is interval censored by $\left(x_{i}-1,x_{i}+1\right)$ is presented in Figure A6. Results are almost identical to the previously presented results. It was also investigated whether the smaller duration of hospitalization for <20 years can be due to the neonati, for which the duration of stay is often determined by duration of post-delivery recovery of the mother. And indeed, the LoS in hospital for the youngest age group increases slightly if we take out the children of 0 years to 4.1(2.2,7.6) days for males and 3.7(2,6.9) days for females. The LoS in hospital for recovered patients increases to 6.4(3.7,11) days for males and 5.9(3.4,10.2) days for females of age between 1 and 19 years of age, making it very similar to the 20–60 years old patients that recovered. No impact was observed on the LoS in ICU.

## 4. Discussion

Previous studies in other countries reported a mean time from symptom onset to hospitalization of 2.62 days in Singapore, 4.41 days in Hong Kong and 5.14 days in the UK [16]. Other studies report mean values of time to hospitalization ranging from 5 to 9.7 days [8,17,18]. In Belgium, the mean time from symptom onset to hospitalization overall is 5.74 days, which is slightly longer as compared to the reported delay in other countries, but depending on the patient population, estimates range between 3 and 10.4 days in Belgium. The time from symptom onset to hospitalization is largest in the working age population (20–60 years), followed by the elderly (60–80) years. If we compare patients within the same age group, it is observed that the time delay is somewhat higher when patients come from a nursing home facility, with an increase of approximately 2 days. The time from symptom onset to diagnosis has a similar behaviour, with a slightly longer delay as compared to time from symptom onset to hospitalization. The diagnosis was typically made upon hospital admission to confirm COVID-19 infection during the first wave, explaining why the time from symptom onset to hospitalization is very close to the time to diagnosis.

To investigate the length of stay in hospital, we should make a distinction between patients that recover or that die. While the median length of stay for patients that recover varies between 5 days (in the young population) to 15.7 (in the elderly), the median length of stay for patients that die varies between 5.7 days (in the elderly) and 12.2 days (in the working age population). In general, it is observed that the length of stay in hospital for patients that recover increases with age, and males need a slightly longer time to recover as compared to females. But, patients living in nursing homes leave hospital sooner as compared to patients in the same age group from the general population. Patients living in nursing homes might be more rapidly discharged from hospital to continue their convalescence in the nursing home, whereas this is probably less the case for isolated elderly patients. In contrast, the time between hospitalization and death is longest for the working age population, with shorter survival time for the seniors and the elderly. The length of stay in hospital for patients that die is longer for patients coming form nursing homes, as compared to patients from the same age group from the general population. A similar trend is observed for the length of stay in ICU.

Over the course of the first wave, the LoS has slightly decreased. This result is corrected for possible bias of prolonged lengths of stay being less probable for more recently admitted patients. Therefore, this might be related to improved clinical experience and improved treatments over the course of the epidemic. But note that also varying patients profiles in terms of comorbidities or severity of disease over time can explain this trend, and it would therefore be interesting to correct for the patient’s profile in a future study. The length of stay in Belgian hospitals is within the range of the once observed in other countries, though especially the length of stay in ICU seems shorter in Belgian hospitals. Reference [19] report a median length of stay in hospital of 14 days in China, and of 5 days outside of China. The median length of stay in ICU is 8 days in China and 7 days outside of China [20]. Reference [1] report estimated length of stay in England for COVID-19 patients not admitted to ICU of 8.4 days and for ICU length of stay of 12.4 days. It should however be noted that the criteria for hospital (and ICU) admission and release might be distinct in the different countries.

Different sensitivity analysis indicated that the results are robust to some of the assumptions made in the modeling. However, alternative methods could still be investigated to improve the estimation of the delay distributions. First, alternative distributions can be used, having more than two parameters and thus more flexibility, e.g., generalized gamma distributions (for which the gamma, exponential and Weibull distributions are special cases). Second, a truncated doubly-interval censored method could be considered to account for the uncertainty in both time points determining the observed delays (and their intervals). Third, there is possible reporting bias in the time of symptom onset, which can influence the results. Finally, the impact of severity of illness and co-morbidity on the length of stay in hospital is very important. This was not investigated in this study as this information was not made available, but is an important factor to investigate in future analyses.

## Figures and Tables

**Table 1:** Summary of the regression of the scale (λ) and shape (γ) parameters for reported delay time between symptom onset and hospitalization and between symptom onset and diagnosis, based on a truncated Weibull distribution: parameter estimate, standard error and significance (* corresponds to *p*-value <0.05; ** to *p*-value <0.01 and *** to <0.001). The reference group used are females of age >80 living in nursing home that are hospitalized in the period 01 March to 20 March.

	log(λ)		1/γ	
	Est	s.e.	Est	s.e.
Symptom onset—hospitalization				
(Intercept)	1.218	0.044 ***	1.208	0.036 ***
Male	0.032	0.020	−0.076	0.017 ***
Age <20	−0.535	0.104 ***	0.033	0.084
Age 20–60	0.618	0.034 ***	−0.490	0.028 ***
Age 60–80	0.433	0.035 ***	−0.251	0.029 ***
Nursing home: No	−0.452	0.044 ***	0.250	0.039 ***
Nursing home: Unknown	−0.046	0.021 *	0.143	0.019 ***
Period 21 March to 30 March	0.234	0.033 ***	−0.100	0.028 ***
Period 31 March to 18 April	0.187	0.035 ***	0.137	0.030 ***
Period 19 April to 12 June	0.028	0.047	0.495	0.039 ***
Symptom onset—diagnosis				
(Intercept)	1.527	0.042 ***	1.232	0.035 ***
Male	0.040	0.019 *	−0.098	0.016 ***
Age <20	−0.400	0.081 ***	−0.196	0.059 ***
Age 20–60	0.383	0.031 ***	−0.389	0.025 ***
Age 60–80	0.284	0.031 ***	−0.201	0.026 ***
Nursing home: No	−0.480	0.038 ***	0.062	0.031 *
Nursing home: Unknown	0.022	0.019	−0.004	0.016
Period 21 March to 30 March	0.181	0.034 ***	−0.187	0.030 ***
Period 31 March to 18 April	0.029	0.036	0.097	0.032 **
Period 19 April to 12 June	−0.297	0.047 ***	0.545	0.042 ***

**Table 2:** Summary of the regression of the log-mean (μ) and log-standard deviation (σ) parameters for the length of stay in hospital and ICU, based on the lognormal distribution: parameter estimate, standard error and significance (* corresponds to *p*-value<0.05; ** to *p*-value <0.01 and *** to <0.001). The reference group used are females of age >80 living in nursing home that are hospitalized in the period 01 March to 20 March. A ‘/’ indicates that this variable was not included in the final model.

	μ		σ	
	Est	s.e.	Est	s.e.
Length of stay in hospital				
(Intercept)	2.480	0.035 ***	0.913	0.024 ***
Male	0.091	0.019 ***	/	/
Age <20	−1.143	0.067 ***	/	/
Age 20–60	−0.553	0.024 ***	/	/
Age 60–80	−0.087	0.023 ***	/	/
Period 21 March to 30 March	−0.242	0.034 ***	−0.008	0.027
Period 31 March to 18 April	−0.270	0.034 ***	0.017	0.027
Period 19 April to 12 June	−0.359	0.038 ***	0.087	0.030 **
Length of stay in ICU				
(Intercept)	2.183	0.152 ***	1.052	0.029 ***
Age <20	−0.443	0.401	/	/
Age 20–60	0.098	0.126	/	/
Age 60–80	0.273	0.122 *	/	/
Nursing home: No	−0.282	0.147	/	/
Nursing home: Unknown	−0.170	0.072 *	/	/
Period 21 March to 30 March	−0.135	0.112	/	/
Period 31 March to 18 April	−0.186	0.115	/	/
Period 19 April to 12 June	−0.418	0.151 **	/	/

**Table 3:** Summary of the regression of the log-mean (μ) and log-standard deviation (σ) parameters for length of stay in hospital for recovered patients and patients that died, based on lognormal distribution and weibull distribution: parameter estimate, standard error and significance (* corresponds to *p*-value <0.05; ** to *p*-value <0.01 and *** to <0.001). The reference group used are females of age >80 living in nursing home that are hospitalized in the period 01 March to 20 March. A ‘/’ indicates that this variable was not included in the final model.

Length of stay in hospital (reason out of hospital = recovered)				
	μ		σ	
	Est	s.e.	Est	s.e.
(Intercept)	2.724	0.044 ***	0.720	0.024 ***
Male	0.077	0.022 ***	/	/
Age <20	−1.074	0.074 ***	/	/
Age 20–60	−0.625	0.032 ***	/	/
Age 60–80	−0.231	0.031 ***	/	/
Nursing home: No	0.261	0.038 ***	0.008	0.031
Nursing home: Unknown	0.012	0.0245	0.099	0.018 ***
Period 21 March to 30 March	−0.307	0.040 ***	0.034	0.027
Period 31 March to 18 April	−0.326	0.039 ***	0.007	0.027
Period 19 April to 12 June	−0.388	0.045 ***	0.097	0.031 **
Length of stay in hospital (reason out of hospital = death)				
	log(λ)		1/γ	
	Est	s.e.	Est	s.e.
(Intercept)	2.573	0.070 ***	0.842	0.014 ***
Age 20–60	0.372	0.084 ***	/	/
Age 60–80	0.162	0.040 ***	/	/
Nursing home: No	−0.383	0.048 ***	/	/
Nursing home: Unknown	−0.160	0.047 **	/	/
Period 21 March to 30 March	−0.142	0.069	/	/
Period 31 March to 18 April	−0.156	0.069 *	/	/
Period 19 April to 12 June	−0.189	0.079 *	/	/

**Figure A1:**
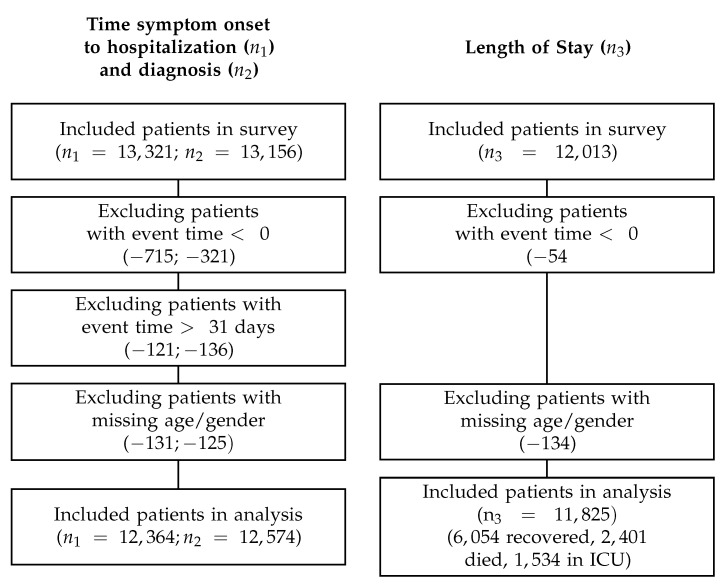
Flow diagram: ($n_{1}$; $n_{2}$) on the left hand side correspond, respectively, to the number of patients for the analysis of time of symptom onset to hospitalization and for time of symptom onset to diagnosis. The number of patients $n_{3}$ on the right hand size correspond to the length of stay in hospital.

**Figure A3:**
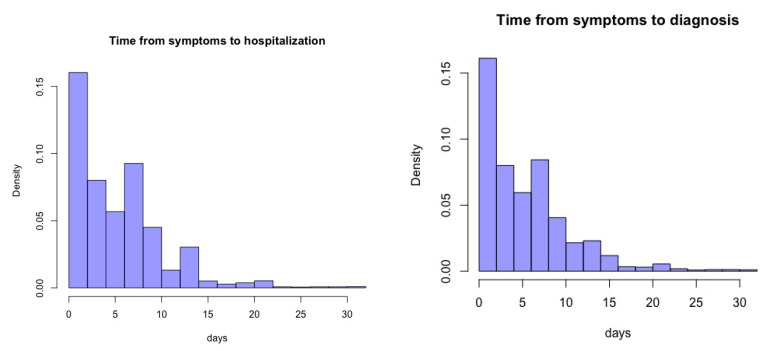

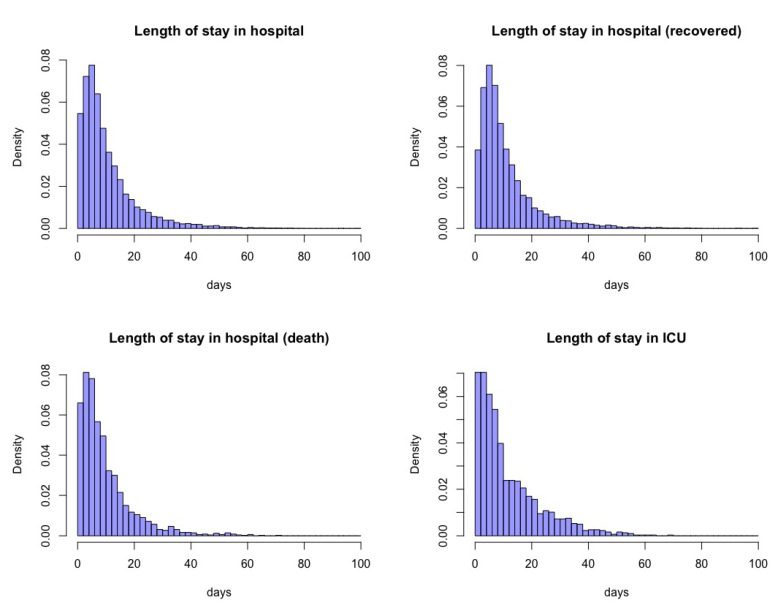
Observed density (proportion of the population in the survey) of time between symptom onset and hospitalization (**top left**) and between symptom onset and diagnosis (**top right**); and observed length of stay in hospital (all COVID-19 patients (**middle left**), recovered patients (**middle right**), patients that died (**bottom left**) and length of stay in ICU (**bottom right**).

**Figure A4:**
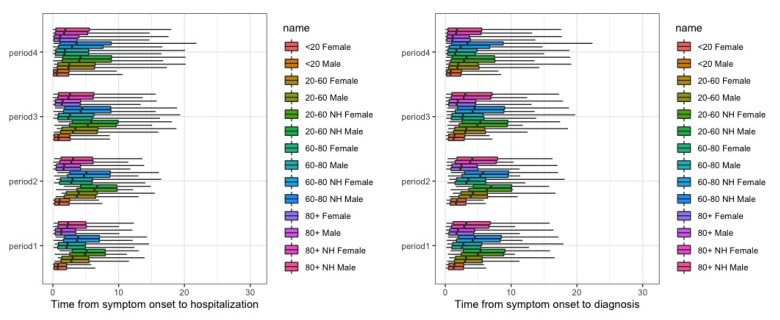
Comparison of delay distribution for different time periods. The boxplots show the estimated 5%,25%,50%,75% and 95% quantiles, based on Weibull regression. Results are based on the time period 01 March to 20 March (period 1), 21 March to 30 March (period 2), 31 March to 18 April (period 3) and 19 April to 12 June (period 4).

**Figure A5:**
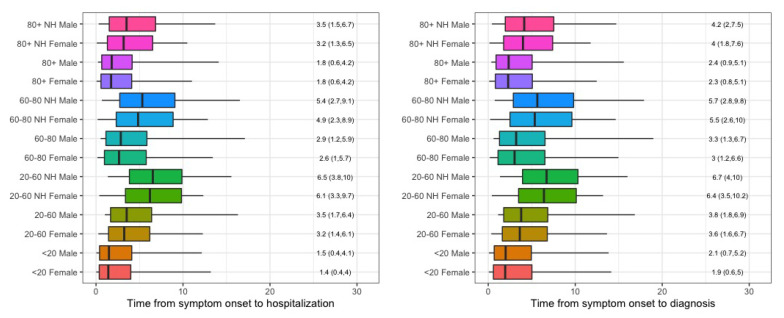
Comparison of delay distribution for different population groups. The boxplots show the estimated 5%,25%,50%,75% and 95% quantiles, based on (untruncated) Weibull regression. Results are based on period 21 March to 30 March.

## Appendix A. (Inclusion/Exclusion Criteria)

A flow diagram of the exclusion criteria is displayed in Figure A1. The time of symptom onset and time of hospitalization is available for 13,321 patients. The date of symptom onset is determined based on the patient anamnesis history made by the clinicians. Patients that were hospitalized before the start of symptoms (i.e., 715 patients) were not included. These include patients with nosocomial infections admitted prior to COVID-19 infection for other long-term pathologies, then got infected at hospital and developing COVID-19-related symptoms long after admission. Patients reporting a delay between symptoms and hospitalization of more than 31 days (i.e., 121 patients) were also not included, because it is unclear for these patients whether the reason for hospital admission was COVID-19 infection. A sensitivity analysis including patients with event times above 31 days is conducted. Patients with missing information on age (i.e., 12 patients) or gender (i.e., 109 patients) were not included in the statistical analysis. This resulted in a total of 12,364 patients which were used to estimate the distribution of the time between symptom onset and hospitalization.

The time of symptom onset and time of diagnosis is available for 13,156 patients. Some of these were diagnosed prior to having symptoms (321) or experienced symptoms more than 31 days before diagnosis (136), and are excluded as these might be errors in reporting dates. Similarly, the delay between symptoms and detection time is truncated at 31 days; but a sensitivity analysis including these patients is performed. In total, 125 patients were removed because of missing information on age and/or gender, resulting in 12,574 patients used in the analysis of the time from symptom onset to diagnosis.

The time between hospitalization and discharge from hospital is available for 12,013 patients, either discharged alive or dead. For patients that were hospitalized before the start of symptoms (i.e., 528 patients), we use the time between the start of symptoms and discharge. Patients with negative time intervals (54 patients) are excluded for further analysis. Another 134 patients were discarded because of missing covariate information with regard to their age or gender. From these patients, we know that 6054 recovered from COVID-19, while 2401 died. From the hospitalized patients, there is information about the length of stay at ICU for 1534 patients.

Note that we analyzed an anonymized subset of data from the hospital COVID-19 clinical surveillance database of the Belgian public health institute Sciensano. Data from Sciensano was shared with the first author through a secured data transfer platform.

## Appendix B. (Patient Characteristics)

**Table A1:** Number of hospitalized patients in the national surveillance survey of COVID-19 hospitalizations, between 1 March 2020 and 12 June 2020.

	Nursing Home			Total
Age	Yes	No	Unknown	
0–19	0 (0.0%)	159 (61.4%)	99 (38.2%)	258
20–59	80 (1.8%)	2506 (58.8%)	1752 (40.4%)	4338
60–79	652 (11.9%)	2585 (47.2%)	2243 (40.9%)	5480
80+	1605 (35.3%)	1562 (34.4%)	1375 (30.3%)	4542
Total	2337 (16.0%)	6812 (46.6%)	5469 (37.4%)	14,618

## Appendix C. (Descriptive Analysis)

The observed distribution of the delay from symptom onset to hospitalization and LoS in hospital are presented in Figure A3. Summary information about these distributions are presented in Table A2 and Table A3.

**Table A2:** Summary of the reported delay time between symptom onset and hospitalization and between symptom onset and diagnosis.

		Mean	Median	2.5%	25%	75%	97.5%	N
Symptom onset—hospitalization								
Overall		5.6	5.0	0.0	2.0	8.0	20.0	12,364
Gender	Male	5.9	5.0	0.0	2.0	8.0	20.0	6700
	Female	5.2	4.0	0.0	1.0	7.0	20.0	5664
Age	<20	3.0	1.0	0.0	0.0	4.0	14.0	227
	20–60	6.8	7.0	0.0	3.0	9.0	20.0	4166
	60–80	5.8	5.0	0.0	2.0	8.0	21.0	4717
	>80	3.9	2.0	0.0	0.0	6.0	16.0	3254
Nursing home	Yes	3.6	2.0	0.0	0.0	5.0	15.0	1863
	No	5.9	5.0	0.0	2.0	8.0	20.0	5885
	Unknown	5.9	5.0	0.0	2.0	8.0	21.0	4605
Period	01 March to 20 March	4.7	4.0	0.0	1.0	7.0	14.0	1365
	21 March to 30 March	6.2	6.0	0.0	3.0	9.0	16.0	4593
	31 March to 18 April	5.6	4.0	0.0	1.0	8.0	21.0	4432
	19 April to 12 June	4.8	3.0	0.0	0.0	7.0	21.0	1974.0
Symptom onset—diagnosis								
Overall		5.8	5.0	0.0	2.0	8.0	20.0	12,574
Gender	Male	6.1	5.0	0.0	2.0	8.0	20.0	6798
	Female	5.5	4.0	0.0	1.0	8.0	21.0	5776
Age	<20	3.5	2.0	0.0	1.0	5.0	13.3	227
	20–60	6.8	7.0	0.0	3.0	9.0	19.0	4131
	60–80	6.1	5.0	0.0	2.0	8.0	21.0	4838
	>80	4.3	3.0	0.0	1.0	6.0	19.6	3378
Nursing home	Yes	3.6	2.0	0.0	1.0	5.0	15.0	1745
	No	6.0	5.0	0.0	2.0	8.0	20.0	6190
	Unknown	6.3	6.0	0.0	2.0	9.0	21.0	4628
Period	01 March to 20 March	5.6	4.0	0.0	2.0	8.0	21.0	1629
	21 March to 30 March	6.6	7.0	0.0	3.0	9.0	17.0	4623
	31 March to 18 April	5.6	4.0	0.0	1.0	8.0	21.0	4427
	19 April to 12 June	4.4	3.0	0.0	0.0	7.0	21.0	1895

**Table A3:** Summary of the reported length of stay in hospital.

		Mean	Median	2.5%	25%	75%	97.5%	N
Length of stay in hospital								
Overall		11.0	8.0	1.0	4.0	14.0	40.0	11,825
Gender	Male	11.3	8.0	1.0	5.0	14.0	42.0	6290
	Female	10.7	8.0	1.0	4.0	14.0	38.0	5535
Age	<20	3.9	3.0	0.0	2.0	5.0	15.0	202
	20–60	8.2	6.0	1.0	3.0	10.0	31.0	3865
	60–80	12.3	9.0	1.0	5.0	15.0	44.0	4425
	>80	12.9	10.0	1.0	5.0	17.0	41.0	3333
Nursing home	Yes	11.5	9.0	1.0	5.0	16.0	34.0	1945
	No	11.1	8.0	1.0	4.0	14.0	43.0	5453
	Unknown	10.6	8.0	1.0	4.0	13.0	40.0	4421
Period	01 March to 20 March	14.7	10.0	1.0	6.0	19.0	53.0	1479
	21 March to 30 March	10.9	8.0	1.0	4.0	13.0	42.0	4214
	31 March to 18 April	10.5	8.0	1.0	4.0	14.0	36.0	4300
	19 April to 12 June	9.2	7.0	1.0	4.0	13.0	29.0	1832.0
Length of stay in ICU								
Overall		12.2	8.0	1.0	4.0	17.0	42.7	1534
Gender	Male	12.7	9.0	1.0	4.0	18.0	43.0	1062
	Female	11.2	7.0	1.0	3.0	15.0	40.2	472
Age	<20	5.2	3.0	1.2	2.0	5.5	17.5	11
	20–60	11.5	8.0	1.0	4.0	16.0	38.5	580
	60–80	13.3	9.0	1.0	4.0	19.0	45.0	796
	>80	9.9	7.0	1.0	3.0	13.0	41.0	147
Nursing home	Yes	8.7	6.0	1.0	3.0	11.2	35.9	96
	No	13.3	9.0	1.0	4.0	19.0	46.0	749
	Unknown	11.5	8.0	1.0	4.0	16.0	41.0	688
Period	01 March to 20 March	14.1	10.0	1.0	5.0	20.0	45.9	284
	21 March to 30 March	12.8	8.0	1.0	4.0	18.0	44.0	691
	31 March to 18 April	11.2	8.0	1.0	4.0	16.0	37.2	434
	19 April to 12 June	8.0	6.0	1.0	3.0	11.0	25.9	125.0

While the observed delay between symptom onset and hospitalization is between 0 and 31 days, 75% of the hospitalizations occur within 8 days after symptom onset. This is however shorter in the youngest age group (<20 years) and in the elderly group (>90 years). Also patients coming from nursing homes seem to be hospitalized faster as compared to the general population. Over the course of the first wave, the observed time between symptom onset and hospitalization was largest in the increasing phase of the epidemic (between 21 March and 30 March). The time between symptom onset and diagnosis is very similar, ranging between 0 and 31 days, with 75% of the diagnoses occurring within 8 days after symptom onset. It should be noted that these observations are based on hospitalized patients, and non-hospitalized patients might have a quite different evolution in terms of their symptoms. As non-hospitalized patients were rarely tested in the initial phase of the epidemic, no conclusions can be made for this group of patients.

The observed median length of stay in hospital is 8 days, with 95% of the patients have values ranging between 1 and 40 days. 25% of the patients stay longer than 14 days in the hospital. The median length of stay seems to increase with age (from 3 days in age group <20 to 6 in age group 20–80, 9 in age group 80–90 and 10 days in age group >90). On the other hand, with time since introduction of the disease in the population, the length of stay seems to decrease, though this might be biased due to incomplete reporting of LOS in patients who are actually still admitted at the time of writing. Therefore, these observed statistics should be interpreted with care. Similar results are observed for the length of stay in ICU. (Figure A4 and Figure A5)

## Appendix E. (Collaborators)

**Table A4:** List of members of the Belgian Collaborative Group on COVID-19 Hospital Surveillance and affiliations.

Name	Institution
Amir-Samy Aouachria	Centre Hospitalier Chrétien, Liège
Kristof Bafort	Mariaziekenhuis, Pelt
Leïla Belkhir	Cliniques Universitaires Saint-Luc, Brussels
Nathalie Bossuyt	Sciensano
Vincent Colombie	Centre Hospitalier Epicura, Baudour
Nicolas Dauby	Centre Hospitalier Universitaire Saint-Pierre, Brussels
Paul De Munter	Universitair Ziekenhuis Leuven, Leuven
Jessika Deblonde	Sciensano
Didier Delmarcelle	Clinique St. Jean, Brussels
Mélanie Delvallee	Centre Hospitalier de Wallonie Picarde, Tournai
Rémy Demeester	Centre Hospitalier Universitaire de Charleroi, Charleroi
Thierry Dugernier	Clinique Saint-Pierre, Ottignies
Xavier Holemans	Grand Hôpital de Charleroi, Charleroi
Frédéric Thys	Grand Hôpital de Charleroi, Charleroi
Benjamin Kerzmann	Clinique Notre Dame de Grâce, Gosselies
Pierre Yves Machurot	Centre Hospitalier de l’Ardenne
Philippe Minette	Centres Hospitaliers Jolimont
Jean-Marc Minon	Centre Hospitalier Régional de la Citadelle, Liège
Saphia Mokrane	Hôpitaux Iris Sud, Brussels
Catherine Nachtergal	Cliniques de l’Europe, Brussels
Séverine Noirhomme	Centre Hospitalier Régional de Namur
Denis Piérard	Universitair Ziekenhuis Brussel, Brussels
Camelia Rossi	Centre Hospitalier Universitaire Ambroise Paré, Mons
Carole Schirvel	CHIREC, Brussels
Erica Sermijn	A.S.Z. Ziekenhuis, Aalst
Frank Staelens	OLV Ziekenhuis, Aalst
Filip Triest	Algemeen Ziekenhuis Sint Lucas, Gent
Dominique Van Beckhoven	Sciensano
Nina Van Goethem	Sciensano
Jens Van Praet	Algemeen Ziekenhuis Sint Jan, Brugge-Oostende
Anke Vanhoenacker	Ziekenhuisnetwerk Antwerpen
Sarah Cooreman	Algemeen Ziekenhuis. Monica
Elise Willems	Algemeen Ziekenhuis Nikolaas, Sint-Niklaas
Chloé Wyndham-Thomas	Sciensano
